# Early life exposure to nicotine modifies lung gene response after elastase-induced emphysema

**DOI:** 10.1186/s12931-022-01956-4

**Published:** 2022-03-03

**Authors:** Sanja Blaskovic, Yves Donati, Isabelle Ruchonnet-Metrailler, Yannick Avila, Dominik Schittny, Christian Matthias Schlepütz, Johannes Constantin Schittny, Constance Barazzone-Argiroffo

**Affiliations:** 1 Department of Pediatrics, Gynecology and Obstetrics, Faculty of Medicine, University of Geneva, 4 rue Gabrielle-Perret-Gentil, 1211 Genève 14, Switzerland; 2 Department of Pathology and Immunology, Faculty of Medicine, University of Geneva, Geneva, Switzerland; 3 Institute of Anatomy, University of Bern, Bern, Switzerland; 4 Swiss Light Source, Paul Scherrer Institute (PSI), 5232 Villigen, Switzerland

**Keywords:** Nicotine, Lung development, Emphysema

## Abstract

**Background:**

Chronic obstructive pulmonary disease (COPD) is among the top 5 causes of mortality in the world and can develop as a consequence of genetic and/or environmental factors. Current efforts are focused on identifying early life insults and how these contribute to COPD development. In line with this, our study focuses on the influence of early life nicotine exposure and its potential impact on (a) lung pulmonary functions, and (b) elastase-induced emphysema in adulthood.

**Methods:**

To address this hypothesis, we developed a model of 2 hits, delivered at different time points: mouse pups were first exposed to nicotine/placebo in utero and during lactation, and then subsequently received elastase/placebo at the age of 11 weeks. The effect of nicotine pretreatment and elastase instillation was assessed by (a) measurement of pulmonary function at post-elastase day (ped) 21, and (b) transcriptomic profiling at ped3 and 21, and complementary protein determination. Statistical significance was determined by 3- and 2-way ANOVA for pulmonary functions, and RNAseq results were analyzed using the R project.

**Results:**

We did not observe any impact of nicotine pre- and early post-natal exposure compared to control samples on lung pulmonary functions in adulthood, as measured by FLEXIVENT technology. After elastase instillation, substantial lung damage was detected by x-ray tomography and was accompanied by loss in body weight at ped3 as well as an increase in cell numbers, inflammatory markers in BAL and lung volume at ped21. Lung functions showed a decrease in elastance and an increase in deep inflation volume and pressure volume (pv) loop area in animals with emphysema at ped21. Nicotine had no effect on elastance and deep inflation volume, but did affect the pv loop area in animals with emphysema at ped21. Extensive transcriptomic changes were induced by elastase at ped3 both in the nicotine-pretreated and the control samples, with several pathways common to both groups, such as for cell cycle, DNA adhesion and DNA damage. Nicotine pretreatment affected the number of lymphocytes present in BAL after elastase instillation and some of the complement pathway related proteins, arguing for a slight modification of the immune response, as well as changes related to general body metabolism. The majority of elastase-induced transcriptomic changes detected at ped3 had disappeared at ped21. In addition, transcriptomic profiling singled out a common gene pool that was independently activated by nicotine and elastase.

**Conclusions:**

Our study reports a broad spectrum of transient transcriptomic changes in mouse emphysema and identifies nicotine as influencing the emphysema-associated immune system response.

**Supplementary Information:**

The online version contains supplementary material available at 10.1186/s12931-022-01956-4.

## Background

Chronic obstructive pulmonary disease (COPD) is, according to WHO, currently the 3rd leading cause of death in the world. Lung emphysema, a form of COPD, is the result of a combination of genetic and epigenetic signatures as well as prenatal and postnatal environmental factors [1]. Among the environmental factors, cigarette smoke (CS) is the main cause of emphysema. CS can induce a pro-inflammatory response by promoting lung epithelial cell-mediated secretion of pro-inflammatory molecules, but also an anti-inflammatory response by suppressing the maturation of dendritic cells and proliferation of T cells [2–5]. Nicotine, the main addictive component of CS, has been suggested to have mainly immunosuppressive properties (summarized in [6]). We previously confirmed that mice exposed pre- and postnatally to nicotine show a decreased transcription of a large amount of lung genes involved in the immune response immediately after birth, and that this effect is no longer present at post-natal day (pnd) 16 [7].

According to the fetal origin hypothesis, first proposed by Barker, events occurring during intrauterine life could leave a signature on the progeny [8, 9]. During human lung development, the pulmonary functions achieve their peak around the age of 20, after which they slowly but constantly decline. Initially, the suggested mechanism of COPD was an accelerated decline of lung functions after the peak as a result of a lung damage [10]. However, an alternative mechanism was proposed recently, suggesting that about a half of COPD patients never reach the expected peak of lung functions and therefore even if the decline of lung functions is normal, COPD will occur earlier in life. This underachievement of the peak of pulmonary functions could be secondary to a prior insult, suggesting an early life origin of respiratory diseases [1, 11]. Hence, identification of both genetic and environmental factors that can induce a specific insult and thereby reduce the “normal” peak of lung functions is crucial for the prevention of COPD. In line with this, we hypothesized that the transient effects induced by in utero nicotine exposure could (a) cause an underachievement of pulmonary functions and (b) prime the offspring to a modified response to emphysema insult during adulthood.

To address these hypotheses, we developed a new two-step model combining a pre- and early postnatal exposure to nicotine with elastase-induced emphysema in adulthood. We exposed mice to nicotine during gestation and lactation until pnd16 and subsequently caused a lung injury by instillation of elastase at the age of 11 weeks [12]. Mice were assessed at ped3 and 21 to study the effect of nicotine pre-exposure on (a) pulmonary functions in the absence of elastase, and (b) early and late progression of lung emphysema. Our results suggest that nicotine pretreatment did not affect lung pulmonary functions. Furthermore, elastase instillation induced similar damage in both the control and nicotine-pretreated mice, as assessed at the macroscopic level and by monitoring pulmonary functions. In particular, we observed a significant decrease in elastance and an increase in inflation volume of the lung and both parameters were insensitive to nicotine pre-treatment. However, we observed an effect of nicotine on pressure–volume loop area. Indeed, in the control, this parameter was significantly increased by elastase whereas in nicotine pre-treated samples this parameter was not changed.

On the other hand, at the transcriptomic level nicotine induced substantial differences, both in the saline and elastase group. In particular, nicotine modified the immune response upon elastase instillation at ped3. While 3 out of the top 10 pathways activated in the control group upon elastase instillation were associated with inflammation and immune response, no such pathways were activated in the nicotine pre-treated samples. Furthermore, immunoglobulin (Ig) response was drastically reduced with nicotine, where Ig accounted only for 0.9% of elastase-induced genes compared to 7.6% in the control samples.

## Materials and methods

### Animals

C57BL/6J mice (*Mus Musculus*) were obtained from the animal facility of University of Geneva. The mice were kept in specific-pathogen-free conditions, with alternating 12 h day/12 h night and access to food and drinking water ad libitum. The treatment with nicotine (200 mg L^−1^) was administered via the drinking water supplemented with 2% saccharin from the beginning of the gestational period until pnd16 days [nicotine group (N)], as previously described [7]. The control group received water with 2% saccharin [control group (C)]. At day 16 post birth, nicotine and saccharin supplementations were stopped and all the mice returned to plain drinking water.

At 11 weeks of age, each control/nicotine pre-exposed mouse was allocated to 1 experimental group, to receive vehicle or elastase instillation. The groups were defined before the intranasal (IN) instillation in order to get a male/female representative from each litter (from control or nicotine exposed group) amongst the vehicle or elastase-instilled group in comparable numbers. In practice, at weaning day, females and males of each litter (obtained under control or nicotine condition) were separated and raised until IN instillation. One day before instillation, mice were weighed and tail-numbered (using non-toxic permanent marker) by random drawing. Then for each individual mouse, allocation to vehicle or elastase instillation was determined by coin toss until completion of each experimental block.

#### Intranasal instillation

Vehicle (sodium acetate (NaOAC), 0.02 M, pH = 5/Glycerol 50%) and elastase (High purity porcine pancreatic elastase, #EC134, Elastin Products, Owensville, MO, USA, stored at − 20 °C, 600U/ml in NaOAC, 0.02 M, pH = 5/Glycerol 50%) were diluted 1/5 in PBS. The calculated respective volumes for vehicle or elastase (0.2 U/g body weight) were intranasally instilled under 5% isoflurane anesthesia as performed previously [9]. For pain prevention, mice received 0.1 mg/kg body weight buprenorphine (Temgesic, Indivior Schweiz AG, CH) by subcutaneous (s.c.) injection 1 h before and 3 h after instillation.

Samples were collected at 2 different time points: ped3 and 21. Lung preparation for further analysis was performed as described previously [13]. Briefly, the airspace of the lung was filled with a solution of 4% paraformaldehyde in phosphate buffered saline (PBS) at a constant pressure of 20 cm water column. At this pressure, the lung reaches roughly its total lung capacity [14]. Lung volume was measured with water displacement method [15]. Finally, we had 4 different groups of animals: control-saline (C-S), control-elastase (C-E), nicotine-saline (N-S) and nicotine-elastase (N-E). Animals from each litter were randomly allocated to each of the 4 groups. We tried as far as possible to respect the equilibrium between female and male representation in each group.

### Lung function measurements

#### Preparation of mice

Mice were anesthetized with 5% sevoflurane inhalation (Sevorane. AbbVie AG, CH) followed by a s.c. injection of metomidate (60 mg/kg, Syndel Laboratories Ltd, Canada) and fentanyl (60 μg/kg, Sintetica SA, CH). After 5 min and a local anesthesia by s.c. injection of 1% xylocaine (0.1 mL, Rapidocain 1%, Syntetica SA, CH), the trachea was exposed and cannulated to mechanically ventilate the mice with a Flexivent system (Emka Technologies, Falls Church, VA, USA) in volume-controlled mode (tidal volume 7 μL/g, 170 breaths/min, FiO_2_ 50%). The mice were then paralyzed with an intraperitoneal injection of rocuronium bromide (5 mg/kg, Esmeron, MSD, CH) to inhibit spontaneous breathing. Body temperature was controlled by a thermal sensor and maintained close to 38 °C with a heating pad. ECG was monitored by using needle electrodes and the PowerLab data acquisition system (ADinstrument, Dunedin, New Zealand).

#### Measurement of respiratory mechanics

An initial deep inflation maneuver was performed allowing the lung alveoli recruitment and inspiratory capacity measurement (DIV; slow inflation to 30 cm H2O with 3 s breath hold). Then, forced oscillatory respiratory mechanics, airway resistance (Raw) and inertance in series with a constant-phase model incorporating tissue damping (G) and elastance (H) were measured with Flexivent system as previously described [16, 17]. Measurements were repeated to collect 3 fitting values for Raw, G and H. A final pv loop maneuver was performed in order to extract pv loop area (hysteresis). At the end of the experiment, the animal was exsanguinated through section of the cava vein, disconnected from the ventilator, the chest opened and PBS instilled via the endotracheal tube with a syringe containing a volume of 30 μL/g body weight (total mouse lung volume [18]). The bronchoalveolar lavage fluid (BAL) was collected as described previously [19] after 3 gentle wash-in and wash-out maneuvers. Cells were counted and the BAL centrifuged at 250×*g* for 5 min at 5 °C. Lungs were fixed with 4% formaldehyde in PBS delivered via the endotracheal tube under 20 cm H_2_O hydrostatic pressure. Afterwards, lungs were further processed for 3-dimensional imaging.

### Efficacy of the elastase instillation

The efficacy of the elastase instillation of the lungs was assessed by high-resolution x-ray tomographic microscopy. Mice were included into our study according to the following rules. While elastase treated lungs had to show enlarged airspaces in at least 4 lobes, in the control lungs no visible damage was accepted.

Lungs were dried by critical point drying [20–22]. Briefly, fixative was exchanged against PBS, followed by a graded series of ethanol (70–100%). Third exchange was done against CO_2_ at 82 bar using a critical point dryer (EM CPD300, Leica, Heerbrugg, Switzerland). Once in supercritical CO_2_, lungs were brought back gently to ambient conditions and mounted in 500 μl Eppendorf tubes (Eppendorf, Schönenbuch, Switzerland) onto SEM-sample holders (standard ½” pin stubs, Plano GmbH, Wetzlar, Germany).

Samples were scanned using the X-ray tomographic microscopy setup at the TOMCAT beamline of the Swiss Light Source (Paul-Scherrer-Institut, Villigen, Switzerland) [23]. Briefly, the quasi-parallel synchrotron X-ray beam was monochromatized to an energy of 12.0 keV and the transmission image of the sample was converted to visible light after a free-space propagation distance of 50 mm by a 17 µm thick LSO:Tb scintillator. The image on the scintillator was magnified 4 times using a high numerical aperture macroscope custom built by Optique Peter (Lentilly, France) [24] and captured using a pco.EDGE 5.5 (PCO AG, Kelheim, Germany) sCMOS camera, resulting in an effective isometric pixel size of 1.625 μm. 3–7 wide-field 360-degree scans were stacked vertically with slight overlaps along the rotational axis [25–27] for each sample to capture its complete volume. Tomographic reconstructions of the individual 3D-datasets were performed in single-distance phase contrast mode [28] (δ = 2e-7, β = 2.8e-10) with the gridrec algorithm [29] using the TOMCAT reconstruction pipeline (Reco Manager) [30], and finally stitched together using the non-rigid stitching algorithm NRStitcher [31].

### BAL cell count

The cell pellet, recovered after BAL, was resuspended in PBS/BSA 1%, and used to prepare cytospin by centrifugation at 750 rpm for 7 min. Then cells were fixed and slides stained with May Grünwald Giemsa. Cytospins were then microscopically scanned (Zeiss Axioscan.Z1). ZEN2 software was used for differential cell counting. To circumvent bias due to inhomogeneous cell distribution, the counts (at least 200 cells) were performed in a rectangle crossing the whole diameter of the circular cytospin. BAL supernatant was used to measure the levels of different proteins by ELISA as described in the following chapter.

### ELISA

Elisa was performed on BAL supernatant and lung tissue extracted proteins collected from 4 conditions (control-saline (C-S), nicotine-saline (N-S), control-elastase (C-E) and nicotine-elastase (N-E)) to assess the expression of 8 different proteins, namely C–C motif chemokine ligand (CCL) 2 (MJE00B, R&D Systems, Inc.), 8 (#LS-F2848, LS Bio) and 24 (#MBS824712, MyBioSource, Inc.), C-X-C motif chemokine ligand 13 (CXCL13) (#EMCXCL13, ThermoFisher Scientific), tumor necrosis factor receptor superfamily member 18 (TNFRSF18) (#LS-F2674, LS Bio), and 3 complement proteins, V-set and immunoglobulin domain containing 4 (VSIG4) (#ELM-VSIG4-1, RayBiotech Life, Inc.), complement factor D (CFD) (#LS-F6130, LS Bio) and complement C3 (C3) (#MBS763294, MyBioSource, Inc.). To extract the proteins, tissue was cut and disrupted for 30′ on ice with Qiagen TissueRuptur (#9002756, Qiagen) in PBS added with Roche cOmplete™ Protease Inhibitor Cocktail Tablets (#04693159001, Roche). Tissue was then sonicated (4 cycles of 10″) on ice, centrifuged for 10′ at 10000 g, 4 °C and the supernatant was collected for protein quantification ELISAs were performed according to manufacturer’s instructions.

### RNA and library preparation, sequencing and read mapping to the reference genome

ReliaPrep™ RNA Cell Miniprep System purification kit (Promega) was used for total lung RNA isolation at ped3 and 21. Two different approaches were used in our protocol: (1) total RNA isolation (for lungs collected at ped3), and (2) isolation of RNA from laser dissected lungs from 2 different regions (emphysematous and normal). Lungs collected at ped21 were instilled with OCT (50% in PBS, frozen and kept at − 80 °C) and prepared for laser microdissection as previously described with minor adaptations [32, 33]. Briefly, up to 5 OCT/PBS frozen lung cryosections (20 μm thickness) by sample were collected on RNAse free 2 μm PEN membrane glass slide (Leica, cat N°11505189). Fixation with absolute ethanol, OCT removal by partial rehydratation (70% ethanol) and tissue coloration with 0.5% cresyl violet in 95% ethanol were performed immediately in the cryostat chamber (− 20 °C) by successive 20 s steps. The staining solution was finally rinsed with ethanol and the slides stored under desiccation in 50 ml tubes with 3 Å-sieves beads at − 80 °C. A Leica LMD microscope (upright DM6500) equipped with a Cryslas laser and a Leica CC7000 camera were used to perform the tissue laser microdissection. Specific tissue fragments were isolated with a UVI 5 ×/0.12 microdissection objective and regions of interest were defined manually with the LAS-AF software (Leica). The isolated fragments were collected in 0.5 ml PCR tubes. For each sample, 15–30 fragments corresponding to a total surface of 26.3 ± 4.5 × 10^6^ μm^2^ were required to extract 6.2 ± 3.6 ng RNA with a RIN = 6.2 ± 1.6.

For ped3 we had 4 different groups: C-S, C-E, N-S and N-E. Three offspring lungs were used in all the groups except for the group N-E where we had 5 samples. For ped21 the saline groups were the same C-S (N = 3 offspring) and N-S (N = 3 offspring) and the elastase groups (C-E and N-E) were divided into 2 subgroups: emphysematous regions (e) and normal regions (n): eC-E (N = 5 offspring), eN-E (N = 4 offspring), nC-E (N = 3 offspring) and nN-E (N = 3 offspring). cDNA libraries were constructed by the Genomic platform of the University of Geneva using the Illumina TruSeq RNA Sample Preparation Kit (CA, USA) according to the manufacturer’s protocol. Due to different quantity/quality of RNA isolated at ped21, these samples were submitted to an additional amplification step (library type: smarter + nextera). Libraries were sequenced using single-end (50nt-long for ped3 and 100nt-long ped21) on Illumina HiSeq4000. FastQ reads were mapped to the ENSEMBL reference genome (GRCm38.96) using STAR version 2.4.0j [34] with standard settings, except that any reads mapping to more than one location in the genome (ambiguous reads) were discarded (m = 1).

### Unique gene model construction and gene coverage reporting

A unique gene model was used to quantify reads per gene. Briefly, the model considers all annotated exons of all annotated protein coding isoforms of a gene to create a unique gene where the genomic region of all exons are considered coming from the same RNA molecule and merged together.

### RNAseq analysis

All reads overlapping the exons of each unique gene model were reported using feature Counts version 1.4.6-p [35]. Gene expressions were reported as raw counts and in parallel normalized in Reads Per Kilobase Million (RPKM) in order to filter out genes with low expression value (1 RPKM) before calling for differentially expressed genes. Library size normalizations and differential gene expression calculations were performed using the package edgeR [36] designed for the R software [37]. Only genes having a significant fold-change of 2 and more (Benjamini–Hochberg corrected p-value < 0.05) were considered for the RNAseq analysis. Further data analysis was performed using MetaCore software (https://portal.genego.com/) and we considered only pathways with -log(pValue) ≥ 3.

### Statistical analysis

Results were expressed as mean ± SD and were analyzed in PRISM by either a 3-way ANOVA (to assess the general source of variation) or a 2-way ANOVA multiple t-test analysis with recommended tukey or sidak correction for determining significance between different groups. The threshold for significance was set at *p* ≤ 0.05.

## Results

Based on the hypothesis that prenatal and early postnatal events leave a signature on the progeny [8, 9], we exposed mice to nicotine during gestation and lactation until pnd16 (control and nicotine) and subsequently caused a lung injury by instillation of elastase at the age of 11 weeks (saline and elastase) [12]. Three and 21 days after elastase exposure the lungs of the mice were analyzed in 4 different groups of animals: control-saline (C-S), control-elastase (C-E), nicotine-saline (N-S) and nicotine-elastase (N-E).

### Effect of elastase instillation

**Fig. 1:**
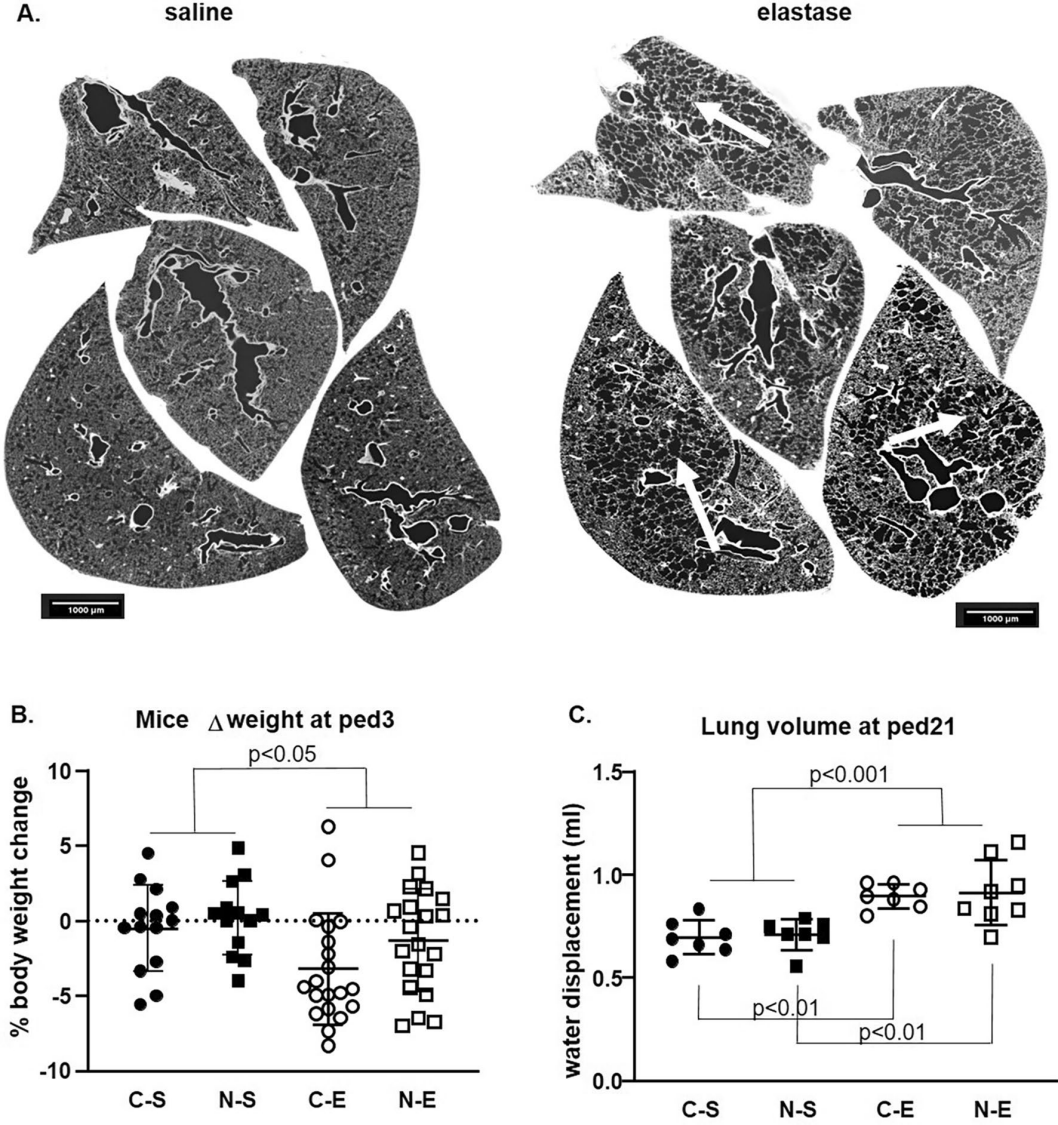
Elastase instillation decreased mouse weight at ped3 and increased lung volume at ped21. The 5 different lobes of saline (control, left) and elastase-instilled (right) lungs were imaged with synchrotron X-ray tomographic microscopy (**A**). White arrows point out the areas with extensive emphysema in elastase-instilled lungs. Weight loss of mice after saline or elastase instillation was measured at ped3 (**B**) and lung volume was measured at ped21 (**C**) for all 4 groups of animals: control-saline (C-S, n = 14 animals for the weight and 7 animals for lung volume respectively), control-elastase (C-E, n = 20 and 7 animals), nicotine-saline (N-S, n = 13 and 7 animals) and nicotine-elastase (N-E, n = 20 and 8 animals). Weight loss was significantly higher in elastase-instilled animals compared to saline-instilled animals (p < 0.05) by two-way ANOVA independently of nicotine pretreatment. No significant difference was present between the 4 groups (**B**). By three-way ANOVA, instillation of elastase significantly affected the lung volume (p < 0.001). Two-way ANOVA analysis between individual groups in both control and nicotine background showed a significant increase of lung volume with p < 0.01 (**C**)

To assess the efficacy of elastase instillation, lungs were imaged on the TOMCAT beamline (Swiss Light Source, Paul Scherrer Institut, Villigen PSI, Switzerland) using synchrotron radiation-based X-ray tomographic microscopy. Imaging revealed that the majority of the mice had developed an extensive emphysema at ped21 (Fig. 1A). However, for the homogeneity of our analysis, we only took into account the mice whose 4 or more lung lobes contained enlarged alveolar spaces. Elastase instillation caused a significant drop in body weight measured at ped3 when both groups (N and C) were analyzed together (Fig. 1B). This difference was not significant within the individual groups. The weight loss was no longer observed at ped21 (data not shown). Lung volume measured at ped21 was also significantly and similarly increased by elastase treatment in groups C-E and N-E. Nicotine treatment did not affect lung volume measurement (Fig. 1C).

**Fig. 2:**
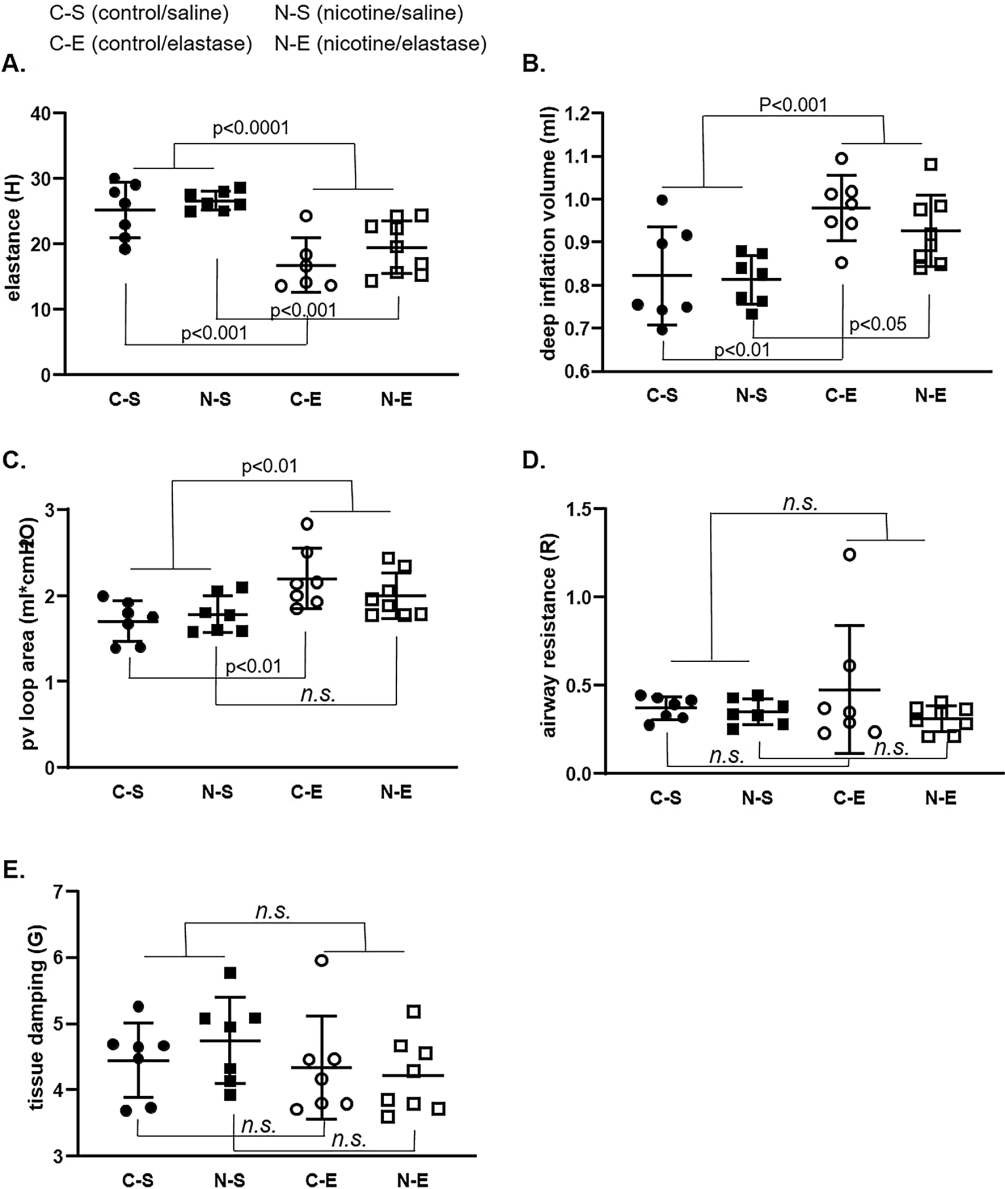
Lung function analysis 21 days (ped21) after elastase instillation. Measures were performed with a FLEXIVENT system. Five different parameters were assessed: elastance (**A**), deep inflation volume (**B**), pressure volume (pv) loop area (**C**), airway resistance (**D**) and tissue damping (**E**). Seven different animals were used in all the groups except in N-E, where we used 8 animals. Elastase instillation significantly affected elastance (p < 0.0001), deep inflation volume (p < 0.001) and pv loop area (p < 0.01) (3-way ANOVA). Analysis between individual groups showed that instillation of elastase significantly decreased elastance and increased deep inflation volume in both control (p < 0.001 and p < 0.01, respectively) and nicotine groups (p < 0.001 and p < 0.05, respectively) and significantly increased pv loop area in the control (p < 0.01), but not in nicotine group (2-way ANOVA). Other parameters were not significantly affected. Control-saline (C-S), control-elastase (C-E), nicotine-saline (N-S), nicotine-elastase (N-E)

We further measured mouse pulmonary functions after saline or elastase instillation (C/N-S and C/N-E). In particular, 5 different parameters were assessed: elastance, deep inflation volume, pv loop area, airway resistance and tissue damping. No significant differences were found for any of the parameters when comparing C-S to N-S. By 3-way ANOVA analysis only elastase instillation (but not nicotine treatment nor gender) had a significant effect on elastance, deep inflation volume and pv loop area (Fig. 2A–C). When comparing individual groups (C-S, N-S, C-E and N-E), we found a decrease in elastance and an increase in deep inflation volume after elastase instillation both in the C-E and N-E groups (Fig. 2A and B). Pv loop area was significantly increased by elastase instillation in the control but not in nicotine samples (Fig. 2C). Airway resistance and tissue damping were not affected by elastase and did not differ in nicotine samples (Fig. 2D and E).

### Gene analysis

To identify the genetic changes induced by nicotine treatment and elastase instillation we performed mRNA analysis of the whole lung at ped3 and ped21. While no gene lists are shown as part of our figures, in our results we report the pathway analysis together with the most affected pathway genes.

**Fig. 3:**
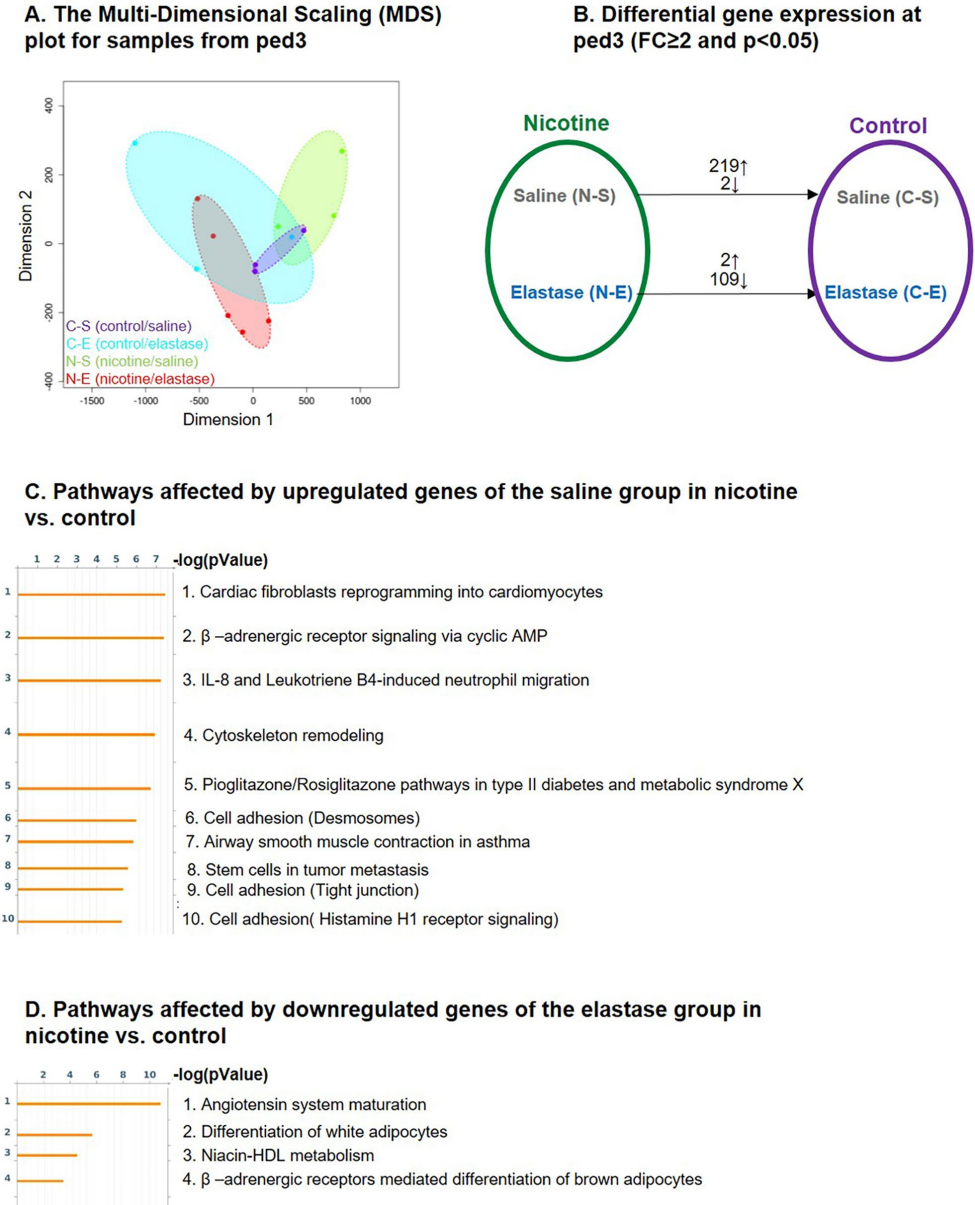
Comparison of gene profiles between control and nicotine-exposed mice with or without elastase instillation at ped3. Lung mRNA was collected at ped3 from 4 different groups, namely control-saline (C-S), control-elastase (C-E), nicotine-saline (N-S), nicotine-elastase (N-E), and sequenced. Three different animals were used in all the groups except in N-E, where we used 5 animals. The data are represented by multidimensional scaling (MDS) (**A**). The distances between the dots for the same color group represent the differences of expression between samples (based on fold changes (FC) between samples). Differential gene expression with significance higher than 0.05 and FC of 2 or higher, of N-S vs. C-S (upper arrow) and N-E vs. C-E (lower arrow) is shown in **B**. Results of pathway analysis include only pathways with -log(pValue) of 3 or more. Analysis was performed on 219 upregulated genes in N-S vs. C-S, and on 109 downregulated genes in N-E vs. C-E. Top 10 pathways are shown for the upregulated genes (**C**), as well as the 4 identified pathways for the downregulated genes (**D**)

**Fig. 4:**
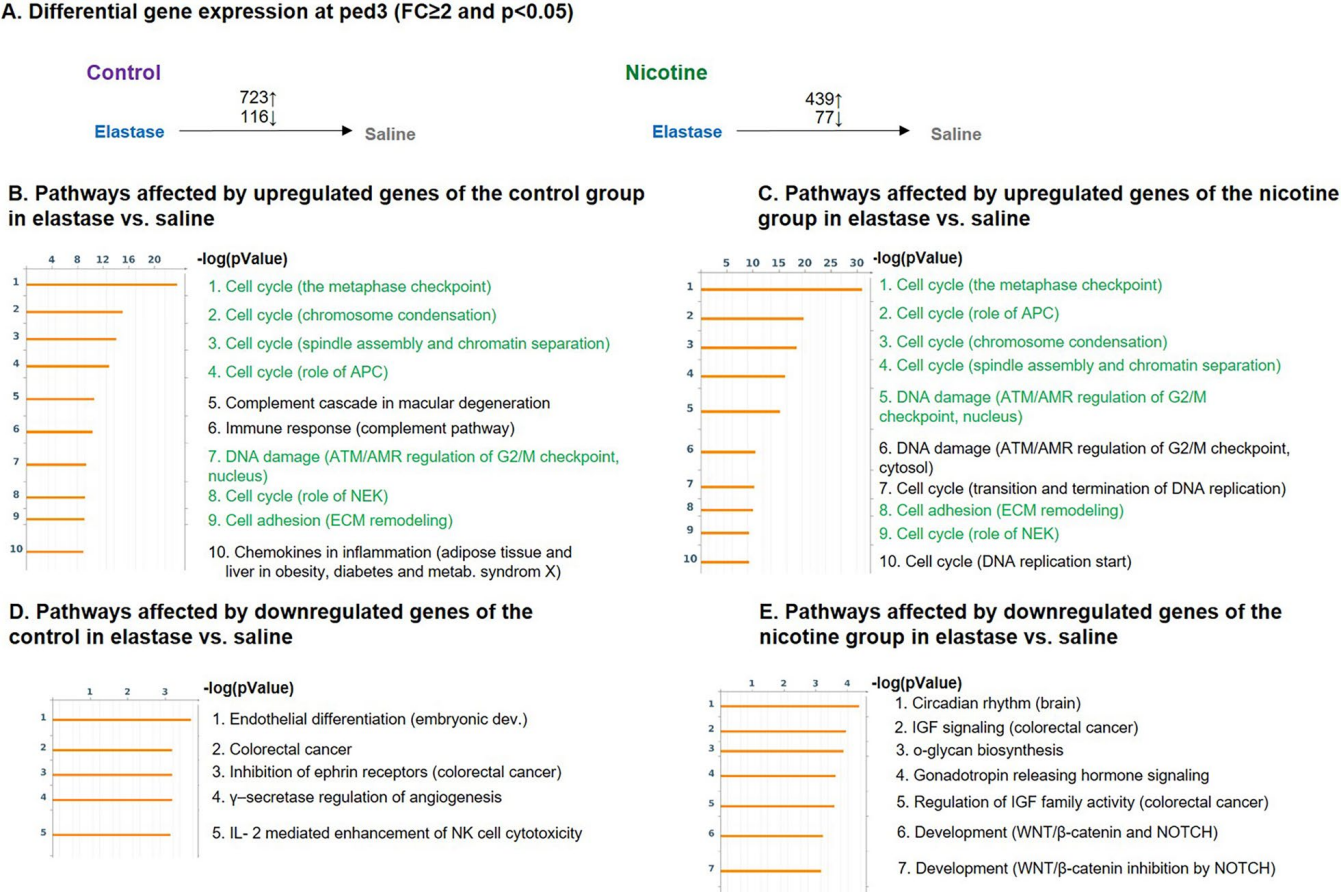
Comparison between elastase and saline instillation in control and nicotine-pretreated mice at ped3. RNAseq analysis, with significance higher than 0.05 and fold changes (FC) of 2 or higher between the elastase and saline treated lungs resulted in 839 (723 upregulated + 116 downregulated) differentially expressed genes in the control group (**A** left) and 516 (439 upregulated + 77 downregulated) differentially expressed genes in the nicotine group (**A** right). Top 10 pathways modified by upregulated genes are shown in **B** for the control and in **C** for the nicotine group. The common pathways between **B** and **C** are shown in green. Top 10 pathways (or less) modified by downregulated genes are shown in **D** for the control and in **E** for the nicotine group. Only pathways with -log(pValue) of 3 or more are shown

**Fig. 5:**
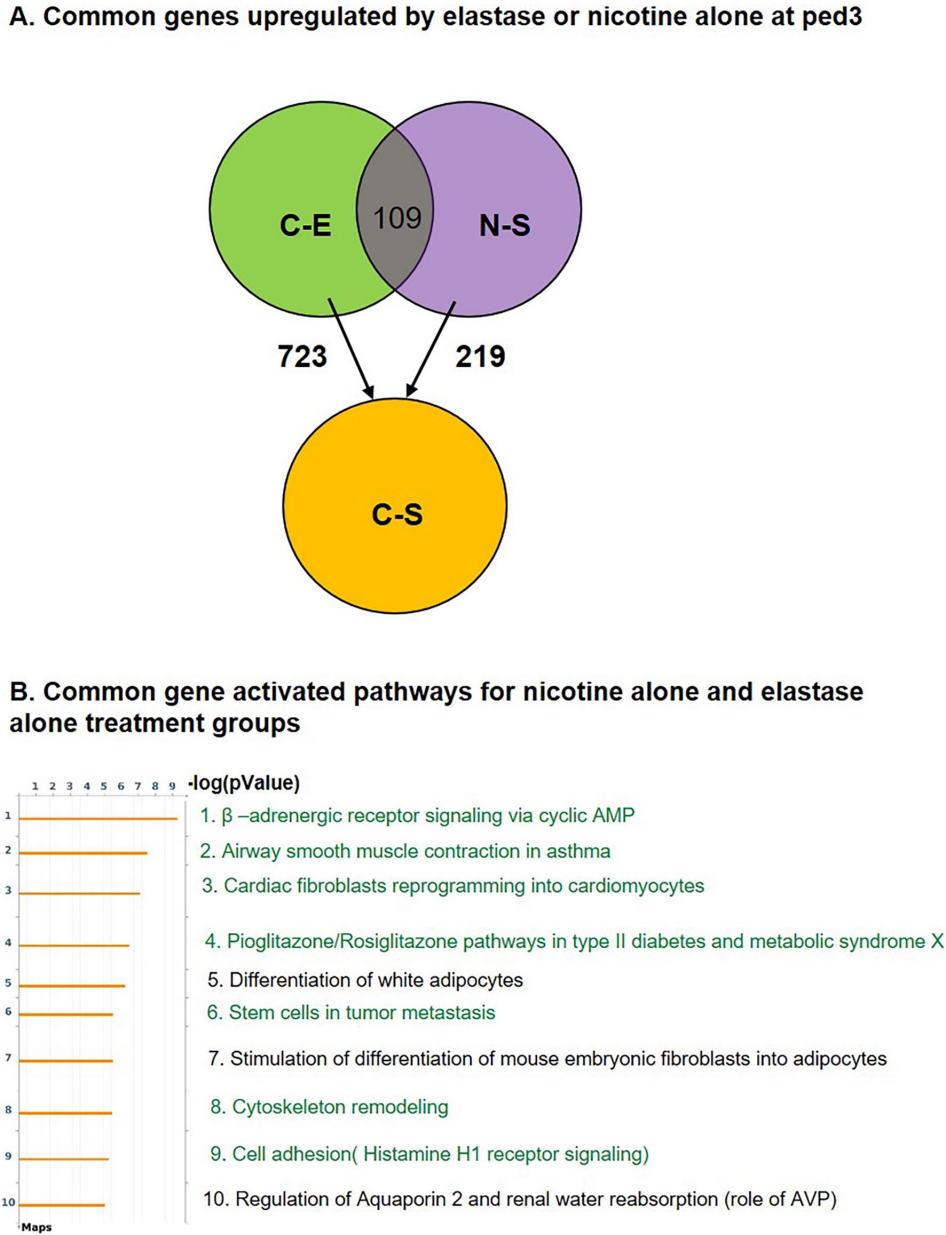
Comparison of genes regulated by elastase instillation or nicotine pre-treatment at ped3. The genetic profile of the control-saline (C-S, orange circle) group was compared either to the control-elastase (C-E, green circle) or to the nicotine-saline (N-S, purple circle) group. Elastase instillation upregulated 723 genes (C-E vs. C-S), while nicotine pre-treatment upregulated 219 genes (N-S vs. C-S) (**A**). The common pool of 109 genes upregulated either by elastase alone (C-E) and nicotine alone (N-S) is shown in **A** gray). Top 10 regulation pathways activated both my C-E and N-S are shown in **B**; the pathways marked in green represent those already detected in the analysis of upregulated genes in the comparison of N-S vs. C-S (shown in Fig. 3C). Adenosine monophosphate (AMP); arginine vasopressin (AVP)

**Fig. 6:**
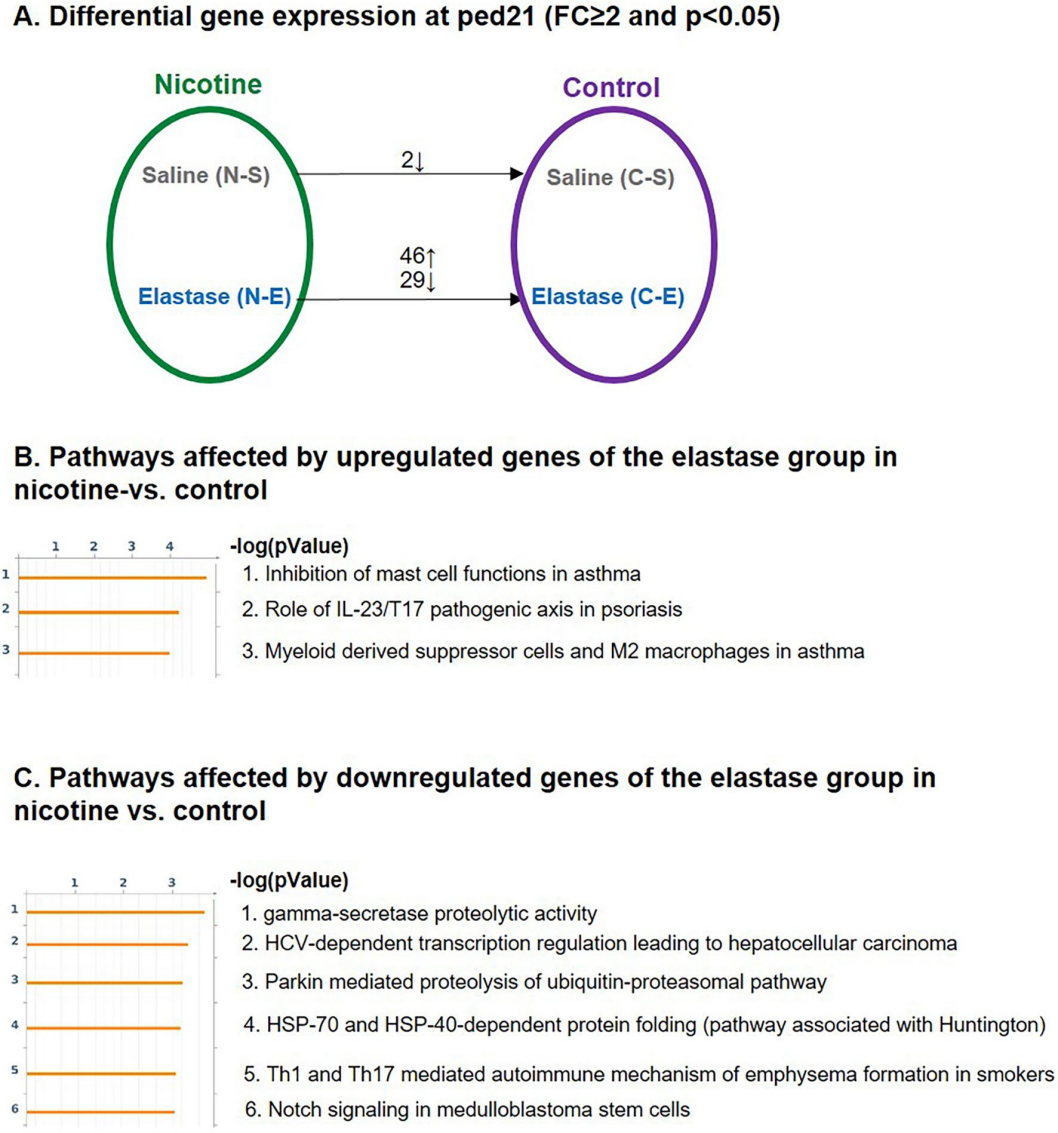
Comparison of gene profiles between control and nicotine-exposed mice with and without elastase instillation at ped21. Genetic profile of 4 different groups, namely control-saline (C-S), control-elastase (C-E), nicotine-saline (N-S), nicotine-elastase (N-E) was compared (**A**). Three to 5 different animals were used in each group. Nicotine exposure resulted in differential expression of 75 genes (46 upregulated and 29 downregulated) in elastase samples (**A** bottom arrow) and only 2 genes in saline samples (**A** upper arrow). Pathway analysis was performed with upregulated (**B**) and downregulated genes (**C**) from the analysis N-E vs. C-E and top 10 (or less) pathways with a -log(pValue) of 3 or more are shown

- Nicotine modifies several genes in control and elastase-instilled lung tissue at ped3
  The variations between the samples are represented in a multi-dimensional scaling (MDS) plot (Fig. 3A). The groups of samples C-S and N-S and the groups C-E and N-E show some overlap. Unlike for the control, where MDS plot showed an overlap between saline (purple oval) and elastase (blue oval) samples, in nicotine pre-treated samples we observed a clear separation between saline (green oval) and elastase (red oval) samples. Nicotine upregulated 219 and downregulated 2 genes in saline background (Fig. 3B).
  The analysis of upregulated genes results in several pathways (Fig. 3C): **cardiac fibroblast reprogramming**^1^, with different actin and myosin genes upregulated from 3 to 8 × and natriuretic peptide A (*nppa*) upregulated 136 × (pathway 1); **β-adrenergic receptor signaling via cyclic AMP** with *perilipin*, *troponin I* and *C*, *phospholamban* and others increased from 3 to 23.5 × (pathway 2); **neutrophil migration** with genes involved in actomyosin fiber remodeling, upregulated 4-9x (pathway 3); **cytoskeleton remodeling** (pathway 4); **metabolism** including upregulation of *adiponectin*, *resistin*, *fatty acid binding protein 1* and genes for several metabolic enzymes from 3 to 40 × (pathway 5); different **cell adhesion associated pathways** with *keratin 5* and *17* upregulated 290 and 245 ×, respectively (pathway 6, 9 and 10), **airway smooth muscle contraction** with genes involved in bronchoconstriction upregulated from 3 to 12.5 × (pathway 7) and **stem cells in tumor metastasis** (pathway 8).
  In elastase treated samples, nicotine upregulated only 2 and downregulated 109 genes (Fig. 3B). The pathway analysis of downregulated genes showed 4 different pathways (Fig. 3D): **angiotensin system maturation**, with *angiotensin* downregulated 6 × (pathway 1), **differentiation of white adipocytes** amongst which the most prominent change was observed for *leptin* that was downregulated 35 × (pathway 2), **Niacin-high density lipoprotein (HDL) metabolism** with HDL, *diacylglycerol O-acyltransferase 2*, *lipase E* and *cytochrome P450 family 2 subfamily E member 1* downregulated from 2.5 to 7 × (pathway 3) and **β-adrenergic receptor mediated differentiation of brown adipocytes** with several genes downregulated from 2.5 to 9 × (pathway 4).
- Effect of elastase-induced emphysema on gene expression
  Instillation of elastase resulted in upregulation of 723 and 439 genes in control (C-E) and nicotine (N-E) lungs, respectively, and downregulation of 116 and 77 genes, respectively (Fig. 4A). Among the top10 pathways detected by differentially expressed upregulated genes, 70% were common to both the control and nicotine background groups. Among these were 5 **cell cycle pathways**, as well as one **cell adhesion** and one **DNA damage pathway** (Fig. 4B and C). The remaining pathways, only present in the control (C-E), were all related to inflammation: **the alternative complement pathway**, with 13 different complement components upregulated from 2 to 85 ×, possibly resulting in increased local inflammation (pathways 5 and 6); and **chemokines in inflamed adipose tissue** responsible for recruitment of macrophages and CD8 + T cells, with 14 genes upregulated from 2 to 16 × (pathway 10) (Fig. 4B). These inflammatory pathways were not found in the nicotine-pretreated lungs and subsequent elastase instillation (N-E).
  In nicotine background the remaining pathways were associated with 2 additional **cell cycle pathways** (pathway 7 and 10), each with 10 different components upregulated up to 11 and 6 × respectively, and **DNA damage response in the cytosol** with 16 different components upregulated from 2 to 14 × (pathway 6) (Fig. 4C). The top 10 pathways detected by genes downregulated by elastase were completely different in control compared with nicotine samples (Fig. 4D and E). Furthermore, the -log(pValue) of these pathways was rather low, unlike the pathways affected by upregulated genes, indicating that only a few genes of the pathways were actually modified. In the control, we found 5 pathways significantly modified with a -log(pValue) of 3 or higher. This includes **endothelial differentiation** with 2 genes, involved in lymphatic and arterial endothelial cell differentiation, downregulated 2 × (pathway 1), 2 pathways involved in **colorectal cancer** (pathway 2 and 3), **γ-secretase regulation of angiogenesis** with *Hey-1* (*Hes Related Family BHLH Transcription Factor With YRPW Motif 1*), a promotor of endothelial cell proliferation, downregulated 3x (pathway 4), and **IL-2 mediated enhancement of NK cell cytotoxicity** with 3 pathway components downregulated 2-3x (pathway 5) (Fig. 4D). Pathways affected with elastase-downregulated genes in nicotine background are shown in Fig. 4E and they encompass **circadian rhythm** with 3 period circadian gene homologs downregulated 2–3 × (pathway 1), **insulin like growth factor (IGF) signaling** (pathway 2 and 5), **o-glycan biosynthesis** with 4 different N- acetylgalactosaminyltransferase-like genes downregulated 2 × (pathway 3), gonadotropin release hormone signaling (pathway 4), and **developmental pathways associated with Wingless-related integration site (WNT)/β-catenin and NOTCH** with *wnt Family Member 10B* downregulated 10x (pathway 6 and 7).
  *Nicotine modifies the immune response in emphysema at ped3*
  In order to investigate the absence of inflammatory pathways at ped3 seen in the transcriptome of nicotine pretreated samples, we analyzed individual genes involved in these pathways (Fig. 4B, pathway 5, 6 and 10) as well as inflammatory markers in BAL. While 7.6% of all genes (55 out of 723) upregulated with elastase in the control were immunoglobulins (Ig) and Ig associated genes, this percentage was drastically reduced to 0.9% (2 out of 219) in nicotine samples, suggesting an impairment of Ig production (Additional file 1: Fig. S1A). We therefore measured the number of different immune cell types in BAL at ped3 (Additional file 1: Fig. S1B–F). Different levels of significance were attributed to elastase by 3-way ANOVA, but not to other tested parameters, such as mouse gender and nicotine. Elastase instillation induced an increase in total BAL cell numbers that was, however, only significant in nicotine samples (Additional file 1: Fig. S1B). The percentage of macrophages was decreased (Additional file 1: Fig. S1C), while the percentages of granulocytes (PMN) and eosinophils were increased with elastase, independently of nicotine treatment (Additional file 1: Fig. S1D and E). Finally, the percentage of lymphocytes increased with elastase only in the control, but not in the nicotine samples (Additional file 1: Fig. S1F). This result is in line with the observed increase of immunoglobulins’ transcription found in the RNAseq analysis of the control samples exposed to elastase.
  To further evaluate the effect of nicotine on the immune system, we tested for the expression of 4 different chemokines (CCL2, 8 and 24, and CXCL13), a TNF family member (TNFRSF18) (Additional file 1: Fig. S2A), as well as 3 proteins from the complement pathway (VISG4, Factor D and C3) (Additional file 1: Fig. S2B), all found modified when comparing RNAseq results from nicotine and the control background of elastase-instilled mice. We detected an upregulation of CCL8 protein in elastase treated mice, independently of nicotine. We did not observe any effect of elastase on the expression of CCL2, CCL24, CXCL13 and TNFRSF18 proteins. However, we found that nicotine decreased the expression of CXCL13 in both saline and elastase samples. For the complement pathway, VSIG4 was upregulated in the control, but not in nicotine-pretreated emphysema samples, and Factor D protein was significantly upregulated in both the control and nicotine emphysema samples. C3 component, found upregulated in the C-E samples of RNAseq, was not modified at the protein level of any of the 4 groups analyzed.
  Finally, analyzing the differences between the control and nicotine groups after elastase or saline treatment we observed that about 15% of genes (109 out of 723) upregulated by elastase in the control were also upregulated in the nicotine only group (Fig. 5A). The top 10 pathways activated by the commonly upregulated genes shared 70% similarity with the pathways upregulated by nicotine alone (Fig. 5B, green colored pathways). This data suggest that nicotine and elastase share some similar gene activation pathways.
- Genetic changes do not persist after elastase-induced emphysema at ped21
  Because the emphysematous regions are not homogenously distributed in the lung, we initially envisioned to study the potential differences between emphysematous and normal-looking regions in elastase-instilled lungs. However, we found the analysis of these multiple regions and pathways difficult to reconcile, and therefore we decided to group the regions for analysis and perform the same analyses as for ped3. To assess if extensive genetic changes, detected at ped3, were permanent or transient, we performed RNAseq analysis at ped21 after the inflammatory phase and once the emphysema had become evident. The comparison between the nicotine and control samples instilled with saline showed minimal differences, with only 2 genes downregulated in nicotine vs. control (Fig. 6A, upper horizontal line, genes *Dynein Axonemal Light Intermediate Chain 1 (Dnali1)* and *Secretoglobin Family 3A Member 1 (Scgb3a1)*). Furthermore, low amounts of genetic changes were observed 21 days after elastase instillation both in the nicotine and control groups, with 1 downregulated gene [*Family With Sequence Similarity 240 Member A (Fam240a)*] and 3 upregulated genes [*Coiled-Coil Domain Containing 78 (Ccdc78), Forkhead Box J1 (Foxj1), GDNF Family Receptor Alpha 1 (Gfra1)*] in the control, and 1 downregulated gene [*Tripartite Motif Containing 58 (Trim58)*] in the nicotine group (data not shown). Hence, the majority of the extensive gene changes detected with elastase instillation at ped3 did not persist until ped21.
- Pro- and anti-inflammatory signals detected in nicotine-pretreated samples after elastase-induced emphysema at ped21
  When comparing both groups instilled with elastase (N-E and C-E), we detected 46 upregulated and 29 downregulated genes in nicotine-pretreated samples. Pathway analysis was performed similarly as for ped3. Both pro-inflammatory and anti-inflammatory signals were detected in nicotine-pretreated samples. Three different immune system-related pathways were significantly affected with the pool of nicotine-induced upregulated genes (Fig. 6B). First, **mast cell function** appears to be inhibited, as 2 receptors involved in Immunoreceptor Tyrosine-based inhibition motifs (ITIM)-dependent inhibitory signaling, *CD22* and *siglec-10*, were both found upregulated. However, one of the genes involved in the cascade leading to the activation of calcium-dependent secretory function of mast cells, *GRB2 Related Adaptor Protein 2* (*grap2*), was also upregulated (pathway 1). The second pathway affected by upregulated genes was the **IL-23/T helper 17 cells (T17) immune system pathway**. Both genes involved in T-cell differentiation (*interleukin 1 beta*) and neutrophil chemotaxis and adhesion (*S100 Calcium Binding Protein A8* (*s100a8* and *a9*)) were found upregulated, which has been reported to lead to chronic inflammation (pathway 2). However, *C–C Motif Chemokine Ligand 20 (Ccl20)*, also a key player in the same pathway and often found upregulated in patients with COPD [38], was downregulated (430x) in nicotine samples. Finally, the third pathway affected by upregulated genes was the one involved in the **regulation of Myeloid derived suppressor cells (MDSC) and M2 macrophages expression** (pathway 3). Increased accumulation of MDSC mRNA suggests a pro-inflammatory environment. The MDSC act as an immunosuppressor and facilitate tumor progression [39].

Analysis of downregulated genes yielded 6 different pathways (Fig. 6C) amongst which only one, Th1- and Th17-mediated autoimmune mechanism of emphysema formation (pathway 5), was associated with an immune response. The levels of 2 components of this pathway, *Ccl20* and *elastin,* were significantly decreased suggesting the inhibitory effect of nicotine pretreatment. In the literature, this pathway has been reported to lead to tissue destruction in emphysema via activation of macrophages that produce/stimulate elastolytic proteases [40].

Other pathways modified with the pool of downregulated genes were not directly associated with the immune system, but some were still reported modified in the COPD patients. **The γ-secretase enzyme** (pathway 1) is involved in the NOTCH signaling pathway and various components of this pathway, including *delta-like canonical notch ligand 1* (*dll1*), have been found downregulated in healthy smokers and smokers with COPD [41]. Our analysis also found that *dll1* was downregulated in N-E vs. C-E samples. The pathway list further encompasses **HCV-dependent transcriptional regulation** (pathway 2), **proteasomal protein degradation** (pathway 3) and **protein folding** (pathway 4) and **notch signaling** (pathway 6). Altogether, these findings suggest a different effect of nicotine-pretreatment at ped21, as compared to ped3.

We finally compared the genes affected at ped3 and ped21 and found a common pool of around 15 genes modified at both time-points (Additional file 1: Table S1). However, when matching for analysis type this number was reduced to 2.

## Discussion

COPD is a complex disease affected by a variety of genetic and environmental factors. Its origins were linked to early aging already in the 1970s [42, 43]. More recently, a stronger emphasis was placed on in utero and early life origins of COPD with the objective of early stage recognition for better prevention and treatment [11]. Hence, it is important to identify genetic changes that can act as early life insults and contribute to the development and progression of COPD. As a potential but not exclusive mechanism, these insults would lead to underachievement of expected pulmonary function whose decline is in all individuals occurring after the age of 22 years [11]. Offspring affected by early insults would hence reach the emphysema-associated decline in lung function earlier than those not exposed to these insults. In this context, our study addresses the effect of in utero and early life nicotine exposure on a potential decline of lung functions as well as the development and progression of elastase-induced lung emphysema.

Lung function values in the saline group were not different between C and N conditions. We did however, find extensive genetic changes induced by nicotine pretreatment at ped3 which did not persist at ped21. The effect of nicotine pretreatment alone, persistent at 11 weeks of age, was somehow surprising, since we previously published that in utero exposure to nicotine caused early (pnd2) extensive but transient changes in gene expression that were no longer detected at pnd16 [7]. Reappearance of transcriptomic changes in nicotine pre-treated saline samples at ped3 either suggests that nicotine effect on gene expression is inconstant or that the process of lung instillation with saline (N-S group) acts as a stressor that can elicit changes in gene expression.

Post-elastase lung analysis showed that our emphysema model was effective since we observed macroscopic lesions in both the control and nicotine pre-treated mice. The functional measurements also confirmed the presence of emphysema in both groups, without substantial differences between mice receiving nicotine in early life and those who did not. As expected, we detected a drop in elastance and an increase in the deep inflation volume (air volume that the lung can take in) [44]. We also found an increase in the pv loop area, an estimate of airspace closure expected to increase in emphysema [45]. On individual group levels, this parameter was, however, only affected in the control and not nicotine pre-treated samples. The fourth measured parameter, airways resistance, was not affected in our study, as already reported in mice and rats [46, 47]. Finally, tissue damping (G), that describes the capacity of energy absorption by the lung, is expected to vary based on the extension of the emphysematous lesions [48]. This parameter should increase in the beginning of emphysema, due to increased tissue density, and drop with the destruction of alveoli [48]. In our study, G was not significantly affected by emphysema, but had a tendency towards a decrease, which is expected for extensive emphysema. These functional and morphometric results confirm the findings reported for mouse emphysema, but give little information about the biological modifications leading to emphysema.

Our transcriptomic analysis revealed a large pool of genes affected by elastase instillation a few days after instillation (ped3). These changes did not persist when emphysematous lesions became apparent (ped21). A similar tendency of time-related decrease in transcriptomic changes was reported in a cigarette smoke-induced emphysema model were a strong inflammatory response was present in the initial acute phase and decreased by half in the emphysematous lung tissue [49]. Our model of emphysema shared some similarities with the CS model. We also detected the upregulation of genes involved in trafficking and accumulation of macrophages, such as chemokine *C–C motif chemokine ligand 2* and *6* and *C–C motif chemokine receptor 2*, reported for both acute and chronic CS exposure. Furthermore, we detected the upregulation of *cd163* (marker of mature tissue macrophages) and *cd14* (involved in development of Th1 response), as found in CS-induced emphysema [49]. On the other hand, the innate immune components, such as toll-like receptors and interferon-related genes, were not affected in our model, while detected downregulated in the CS model. Besides the immune system, different gene groups were affected at different time points in the CS-emphysema model, namely genes involved in drug metabolism, cell survival and apoptosis, extracellular matrix (ECM) maintenance, ubiquitin proteasome system and growth factors [49]. In our study, the majority of these systems were either completely unaffected or only a few genes were modified (for example in the drug metabolism category). We did, however, observe a strong upregulation of ECM maintenance genes (*matrix metallopeptidase*, serpin family members, *cathepsins*, *serine protease 22*, *elastin*, *lysyl oxidase*), as reported for the CS model, but we also detected a predominant effect on cell cycle pathways and damage-induced cell cycle G2/M checkpoint. This may have been due to the acute injury caused by elastase instillation, whereas the CS model is certainly representative of a more chronic model. These data suggest that while the 2 models share some similarities, such as pro-inflammatory signals and high levels ECM remodeling, they also activate different pathways.

While many pathways modified by elastase-induced genes were common to the control and nicotine groups (cell cycle and DNA repair mechanisms), we could still pinpoint major differences, the most striking of which was nicotine-induced downregulation of the immune system. This is particularly interesting in the context of repeated inflammatory signals being the cause of tissue damage, remodeling and repair that alters lung tissue elasticity and robustness and makes it more susceptible to disease [50]. Indeed, in our analysis of the control group, 30% of the top 10 pathways were associated with increased inflammation and immune response after elastase instillation. No such pathways were detected in the nicotine group, hinting towards a decreased immune response, which was already reported in nicotine-exposed lungs by others and us [7, 51, 52]. When assessing the effect of nicotine on the expression of proteins associated with these inflammatory pathways (Fig. 4, pathways 5, 6 and 10), we identified chemokine CXCL13 and complement pathway protein VSIG4 as a potential source of differences caused by nicotine. We also found an increases expression of CCL8 and CFD proteins caused by elastase, but independent from nicotine.

The amount of immunoglobulin genes was greatly reduced in the presence of nicotine. While in the control samples 7.6% of elastase-induced upregulated genes belonged to the immunoglobulin family, in the presence of nicotine, immunoglobulin genes represented only 0.9%, strongly suggesting an impairment of immunoglobulin production. This result was also supported by the absence of increase in BAL lymphocytes percentage upon elastase treatment in nicotine pre-treated samples. The effect of nicotine on the immune system persisted until ped21, where nicotine elicited both pro- (upregulation of IL-23/T17 immune system pathway) and anti-inflammatory effects (upregulation of MDSC and M2 macrophages and downregulation of Th1 and Th17 mediated autoimmune mechanism). Whether a nicotine-induced effect on the immune system can be beneficial or harming in lung emphysema requires further studies. However, it is clear that nicotine-pretreated mice show a different immune response when exposed to a second stressor.

Not surprisingly, due to its ability to bind to acetylcholine receptors on different body organs, nicotine had a strong effect on body metabolism, suggesting a more general rather than local effect and only lung related. Animal studies in rats have suggested previously that early exposure to nicotine could affect metabolism and thereby contribute to development of obesity and type 2 diabetes [53–55]. In our study, nicotine affected the levels of *leptin*, a gene involved in both metabolism and immune response. In literature, systemic and airway concentrations of leptin have been correlated with severity of COPD [56, 57]. We found that nicotine upregulated *leptin* 70x, but subsequent addition of elastase did not further elevate the expression of *leptin*. On the other hand, the level of *leptin* in the controls instilled with elastase was 700 × higher when compared to saline samples, suggesting that nicotine pretreatment predisposes the offspring to a different *leptin*-mediated response in the emphysema model. Nicotine also decreased the expression of genes in the angiotensin system normally expressed in both the lung and white and brown adipose tissue where they regulate metabolism and insulin sensitivity and participate in the immune response [58, 59]. Also, increased activity of angiotensin system was associated with the progression and pathogenesis of COPD [60], hence emphasizing the importance of nicotine effect on this system.

In fact, nicotine and elastase independently activated a common pool of 109 genes (Additional file 2: Table S2 and Fig. 5) and shared many pathways, including metabolic (e.g., insulin resistance), cytoskeleton remodeling- and cell adhesion-associated pathways, all of which have been reported to be modified in COPD patients [61–63]. Because nicotine activates several genes that are also independently activated by elastase alone, the addition of elastase to a nicotine group did not further affect these genes, leading to a bigger difference between the lists of differentially expressed genes after elastase instillation in the nicotine vs. control groups. The significance of such an effect of nicotine pre-treatment is unclear and requires further investigation.

Extensive emphysema-associated genetic changes detected at ped3 were lost by ped21, with only 4 and 1 differentially expressed genes when comparing elastase to saline in control and nicotine conditions, respectively. In a previous study, we observed the same pattern of initial extensive genetic changes in pups’ lungs exposed to prenatal nicotine that rapidly subsided after birth [7]. Due to the small number of differentially expressed genes at ped21, not much overlap was detected between ped3 and 21. Some overlaps were, however, detected when comparing N-E vs. C-E at ped21. Fifteen genes were modified in this comparison, of which half were involved in the immune system response, and already affected at ped3 (Additional file 1: Table S1).

## Conclusions

In conclusion, while our model of elastase-induced emphysema might not fully recapitulate all the mechanisms of the model of CS-induced emphysema nor what occurs in human COPD, it does show the same tendency of early activation of pro-inflammatory signals that slowly decrease over time. Our results do not suggest an effect of early nicotine exposure on adult lung functions and therefore do not support the hypothesis that nicotine alone is a sufficient stressor for an underachievement of the peak of pulmonary functions. Nevertheless, we still identified prenatal nicotine exposure as a significant early insult, affecting both the immune system response and the general metabolic pathways when mice develop pulmonary emphysema. These results pave the way and open perspectives for a better understanding of the pathophysiology of genetic changes caused by early life insults.

## Supplementary Information

**Additional file 1:** Effect of nicotine pretreatment and elastase instillation on the immune response.

**Additional file 2:** Shared pool of genes independently actived by nicotine or elastase.

## Data Availability

The datasets generated and/or analysed during the current study are available in the GEO repository under the accession number GSE167569 (secure token for the reviewers: wtytsiqydlmlnmr).

## Abbreviations

AMP: Adenosine monophosphate
AVP: Arginine vasopressin
BALF: Bronchoalveolar lavage fluid
C3: Complement C3
*Ccdc78*: Coiled-coil domain containing 78
CCL2/8/24: C–C motif chemokine ligand 2/8/24
C-E: Control-elastase
CFD: Complement factor D
COPD: Chronic obstructive pulmonary disease
CS: Cigarette smoke
C-S: Control-saline
CXCL13: C-X-C motif chemokine ligand 13
*Dlli*: *Delta like canonical Notch ligand 1*
*Dnali1*: Dynein axonemal light intermediate chain 1
ECM: Extracellular matrix
FC: Fold change
*Foxj1*: Forkhead box J1
*Gfra1*: GDNF family receptor alpha 1
*Grap2*: GRB2 related adaptor protein 2
HDL: High density lipoprotein
Ig: Immunoglobulin
IGF: Insulin like growth factor
IL: Interleukin
IN: Intranasal
MDS plot: Multi-dimensional scaling plot
MDSC: Myeloid derived suppressor cells
NaOAC: Sodium acetate
N-E: Nicotine-elastase
*nppa*: Natriuretic peptide A
N-S: Nicotine-saline
PBS: Phosphate buffered saline
ped: Post-elastase day
pnd: Post-natal day
pv loop area: Pressure–volume loop area
RPKM: Reads per kilobase million
s.c.: Subcutaneous
*S100A8/A9*: S100 calcium binding protein A8/A9
*Scgb3a1*: Secretoglobin family 3A member 1
T17: T helper 17
TNFRSF18: Tumor necrosis factor receptor superfamily member 18
*Trim58*: Tripartite motif containing 58
VSIG4: V-set and immunoglobulin domain containing 4
WNT: Wingless-related integration site

## Footnotes

1 Significant pathway maps (top 10 or less) obtained by the GeneGo pathway maps analysis are shown in bold letters.
