# Supplementary material for: Early life exposure to nicotine modifies lung gene response after elastase-induced emphysema
Source: Respir Res. 2022 Mar 3;23:44. doi: 10.1186/s12931-022-01956-4 (PMC8895880; doi:10.1186/s12931-022-01956-4)
Supplement: Supplementary file 1 — Additional file 1: Effect of nicotine pretreatment and elastase instillation on the immune response. [file 12931_2022_1956_MOESM1_ESM.docx]

# Early life exposure to nicotine modifies lung gene response after elastase-induced emphysema

Sanja Blaskovic^1,2,3*^, Yves Donati^1,2*^, Isabelle Ruchonnet-Metrailler^1,2^, Yannick Avila^1,2^, [Dominik Schittny](https://pubmed.ncbi.nlm.nih.gov/?term=Schittny+D&cauthor_id=32708256)^3^, [Christian Matthias Schlepütz](https://pubmed.ncbi.nlm.nih.gov/?term=Schlep%C3%BCtz+CM&cauthor_id=31434878)^4^, Johannes C. Schittny^3^, Constance Barazzone-Argiroffo^1,2^

^1^Department of Pediatrics, Gynecology and Obstetrics, Faculty of medicine; University of Geneva, Switzerland; ^2^Department of Pathology and Immunology, Faculty of medicine, University of Geneva, Switzerland; ^3^Institute of Anatomy, University of Bern, Switzerland; ^4^Swiss Light Source, Paul Scherrer Institute, 5232, Villigen PSI, Switzerland.

*Equal contribution

Abbreviated title: Altered gene response in emphysema model after nicotine exposure

Keywords: nicotine, lung development, emphysema, (3-10 max)

Corresponding author:

Constance Barazzone-Argiroffo

Department of Pediatrics, Gynecology and Obstetrics

4 rue Gabrielle-Perret-Gentil
CH - 1211 Genève 14

[Constance.Barazzone@hcuge.ch](mailto:Constance.Barazzone@hcuge.ch)

**Additional Information**

**RESULTS**

**Additional Figure S1:** Effect of early life nicotine exposure on the immune response in emphysema.


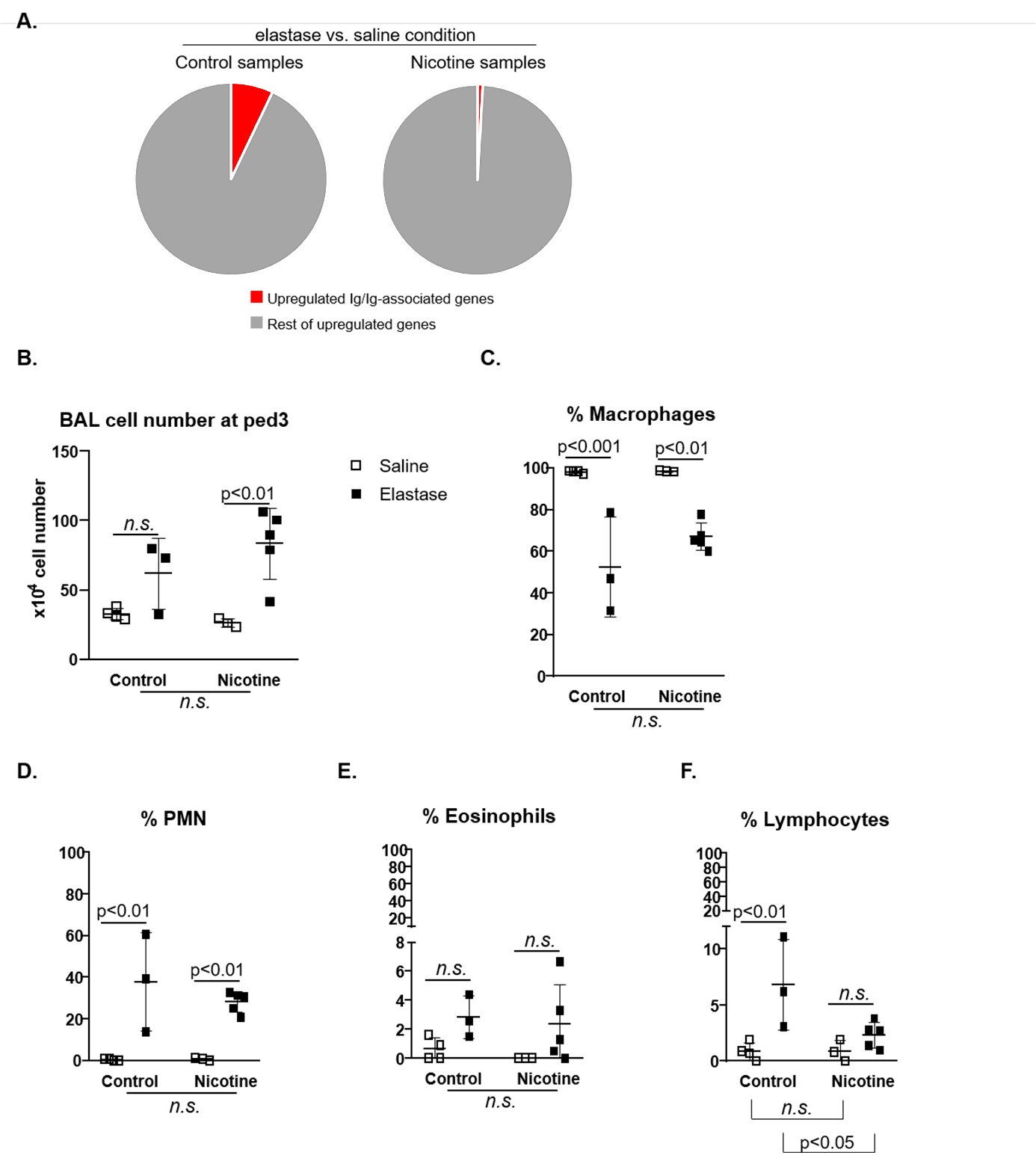


**Legend:** Immunoglobulin (Ig) production was compared between elastase and saline conditions in both control and nicotine-pretreated mice. The fraction of Ig and Ig-associated genes induced by elastase is shown in red (A). Broncho-alveolar lavage (BAL) from ped3, subjected to one of four different conditions (control-saline (C-S), nicotine-saline (N-S), control-elastase (C-E) and nicotine-elastase (N-E)) was assessed for total cell number (B) as well as the number of individual immune cell types: macrophages (C), polymorphonuclear (PMN) cells (D), eosinophils (E) and lymphocytes (F). Significance attributed to elastase by 3-way ANOVA is p<0.05 for (E), p<0.01 for (B) and (F), p<0.001 for (D) and p<0.0001 for (C) (data not shown in the image). Significance for comparison between saline and elastase conditions calculated by multiple comparison tests in both groups is shown with p<0.05, p<0.01, p<0.001 and n.s. (nonsignificant). Comparison between control and nicotine condition in the saline and elastase groups was only significant for the lymphocyte number (p<0.05) in the elastase-instilled group (F).

**Additional Figure S2: Effect of nicotine pretreatment and elastase instillation on the expression of chemokines and complement proteins in bronchoalveolar lavage**

**Legend:** Expression of chemokine ligands CCL2, CCL8, CCL24; CXCL13 and TNF receptor superfamily member 18 (A) as well as three complement pathway components VSIG4; factor D and C3, was accessed by Elisa in BAL taken at ped3 from mice from the different groups (control-saline (C-S), nicotine-saline (N-S), control-elastase (C-E) and nicotine-elastase (N-E)). Significance for comparison between saline and elastase conditions in both groups (control and nicotine) is shown with p<0.05, p<0.01, p<0.001, p<0.0001. Comparison between control and nicotine condition in the saline and elastase groups was only significant for CXCL13 protein. All the other comparisons were not significant (not labelled in the graph)

**
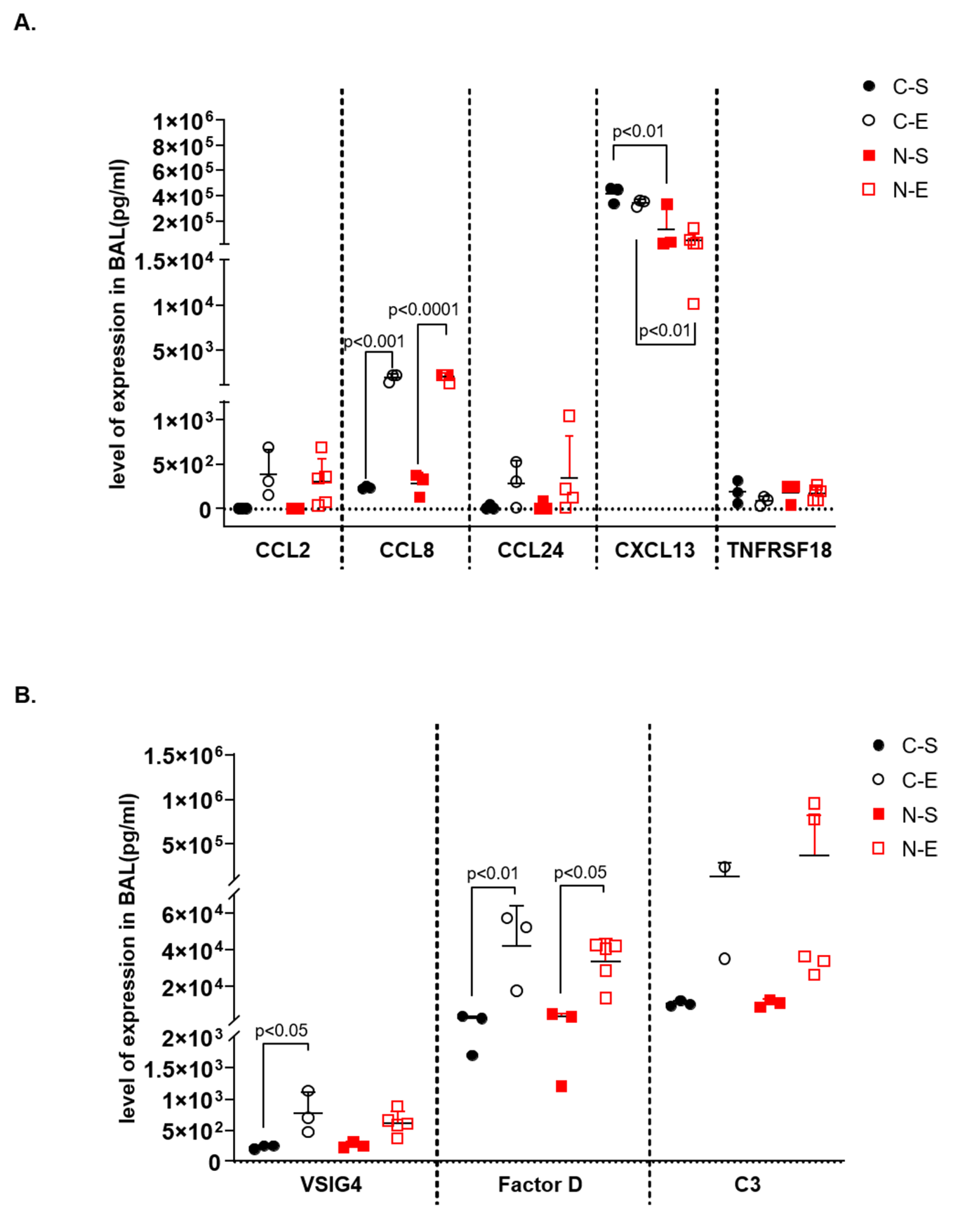
**

**Additional Table S1: Common differentially expressed genes at ped3 and ped21**

| Gene name | Gene description | FC at ped21 (N-E vs. C-E) | FC at ped3  (N-E vs. C-E) | FC at ped3  (N-E vs. N-S) | FC at ped3 (C-E vs. C-S) | FC at ped3 (N-S vs. C-S) | Function |
| --- | --- | --- | --- | --- | --- | --- | --- |
| Dbp | D site albumin promoter binding protein | +5 |  | -5 | -4 |  | hematopoiesis |
| S100A8 | S100 calcium binding protein A8 | +4 |  | +2 |  |  | immune response |
| S100A9 | S100 calcium binding protein A9 | +4 |  | +2 |  |  | immune response |
| Slfn4 | schlafen 4 | +3 |  | +2 |  |  | cell growth |
| Retnlg | resistin-like gamma | +3 |  | +2 |  |  | immune response |
| Ifi27l2a | interferon, alpha-inducible protein 27 like 2A | +2.5 |  |  | -5 |  | immune response |
| Nr1d2 | nuclear receptor subfamily 1, group D, member 2 | +2 |  | -2 |  |  | circadian rhythms and carbohydrate and lipid metabolism |
| Per3 | period circadian clock 3 | +2 |  | -2 |  |  | cyrcadian rythym |
| Mzb1 | marginal zone B and B1 cell-specific protein 1 | +2 | -5 |  | +5 |  | immune response (B cells marker) |
| Wfdc17 | WAP four-disulfide core domain 17 | +2 | +5 |  | +15 |  | immune response (differentiation of B-cells into Ig- secreting cells) |
| Reg3g | regenerating islet-derived 3 gamma | -25 |  |  | +45 | +50 | immune response |
| Tubb2 | tubulin, beta 2B class IIB | -8 |  |  | -3 |  | organization of cell structure |
| Disp2 | dispatched RND tramsporter family member 2 | -3 |  | +2.5 |  | -2 | GPCR signaling and proteasomal degradation |
| Fscn1 | fascin actin-bundling protein 1 | -3 |  | +2 | +3 |  | tumor cell invasion/metastasis |
| Eln | elastin | -2.5 |  | +5.5 | +3 |  | tissue elasticity |

**Legend:** List of genes that were expressed both at ped3 and ped21. The respective fold change (FC) values are reported with a plus sign if the gene was upregulated and a negative sign if the gene was downregulated.

**Additional Table S2:** added as an excel file.
